# Impact of prolonged social crisis on resilience and coping indicators

**DOI:** 10.1371/journal.pone.0305542

**Published:** 2024-08-01

**Authors:** Hadas Marciano, Shaul Kimhi, Yohanan Eshel, Bruria Adini

**Affiliations:** 1 Stress and Resilience Research Center, Tel-Hai College, Tel Hai, Israel; 2 Institute of Information Processing and Decision Making (IIPDM), University of Haifa, Haifa, Israel; 3 ResWell Research Collaboration, Tel Aviv University, Tel Aviv, Israel; 4 Psychology Department, University of Haifa, Haifa, Israel; 5 Head of the Department of Emergency Management and Disaster Management, School of Public Health, Sackler Faculty of Medicine, Tel Aviv University, Tel Aviv, Israel; St John’s University, UNITED STATES

## Abstract

The current study examines, longitudinally, (i.e., on the same sample), to what extent an acute political/social crisis in Israel affected the resilience, distress, and additional psychological indicators of the Jewish population, along with three repeated measurements: The first was conducted shortly before the last elections (in October 2022), the second in February 2023, about two and a half months after the elections, following the formation of a right-wing government, and the third measurement about nine months after the election (August 7–10, 2023). The main results indicated the following: (a) the mean societal resilience among coalition voters increased significantly throughout the three measurements, while it declined significantly among opposition voters. (b) significant differences were identified between coalition and opposition voters, mostly at T2 and T3: opposition supporters reported significantly lower levels of societal resilience and hope, and higher levels of distress symptoms and sense of danger, compared to those of coalition supporters. We concluded that the continued social/political conflict in Israel is multidimensional and impacts diverse areas such as values, perspectives, and supporting as well as suppressing coping indicators. The differences between the two voter groups may be primarily the result of political radicalization and polarization processes, that aim to widen gaps to achieve political power. As many countries are currently facing acute political crises and similar radicalization, similar studies should be conducted in varied societies to investigate the generalizability of the findings.

## Introduction

The recent social/political unrest in Israel has been brewing for several years, fuelled by a series of indecisive elections that failed to produce a stable, long-term government. This period of instability has been marked by intense public discourse on the principle of ’separation of powers’ within the country, a critical issue highlighted by former research [1]. In Israel, the government is composed of people whose parties were elected to the parliament (the Knesset). This practice has several implications: 1) a parliamentary majority for the government is almost guaranteed in practice; 2) it grants small or sector-specific parties significant sway in government’s formation and decision-making; and 3) the separation of powers is blurred between the legislative and executive branches, as both are drawn from the same elected officials. Maor et al. (2020) argue that this confluence of factors, along with ideological polarization and contentious political atmosphere, has led to numerous petitions being filed with the Israeli High Court of Justice (HCJ) [1]. Consequently, the Israeli HCJ has found itself embroiled in what many consider to be political matters. Over the years, this has sparked a public debate around the notion that the judiciary may be circumventing the will of the populace. This debate touches on core issues of democratic governance and the balance of power, highlighting a critical juncture in Israel’s political landscape where the role and reach of judicial intervention in political affairs are being questioned.

On November 1^st^, 2022, elections were held in Israel, after which a government was formed, based on right-wing parties. The elections were held after a prolonged political crisis that the State of Israel experienced from the middle of 2019 until the end of 2022. During this crisis, five rounds of elections were held in less than three years, due to the inability of any of the political parties to establish and sustain a stable government [2]. The results of the November 2022 election enabled the formation of a coalition that appeared to be stable (with a majority of 64 out of 120 seats in the parliament). However, soon after the government was established it expedited the process of initiating laws that the coalition parties referred to as "judicial reform", while the opposition parties and many of the protesting Israelis saw as the government’s attempt to “reshape the Israeli polity, the principles on which Israeli culture and society were established, and perhaps even the most fundamental tenets of Zionism” [3, p. 1]. Thus, this judicial reform may be viewed by these individuals as shattering the accepted rules of the political governance systems or in other words a "coup d’état" [4]. Israel’s democracy is widely regarded, by both scholars and the general populace, as delicate and fragile, constantly under threat of undermining its liberal-democratic foundations. Notable works underscore this perspective, highlighting the precarious nature of Israel’s political system [5, 6]. This fragility precipitated a societal crisis in Israel that persisted for many months casting a shadow over the nation’s governance and public discourse. The crisis, which had gripped the country and showed no signs of abating, was abruptly interrupted by a Hamas attack on Israel’s southern region (on October 7^th^). The crisis was accompanied, among other things, by substantial demonstrations that were held over a prolonged period [7]; the extensive protests against the government took place every weekend since December 2022 (up to October 7^th^ they continued for 39 weeks). According to political experts, the scale of the protests and the length of time they lasted are unprecedented in Israel’s history [3, 8].

The origins of the current crisis are ingrained in the distant past and were apparent even during the establishment of the state of Israel in 1948 [e.g., 9]. However, it seems that the crisis has reached its current extremely high level due to the last election and the current coalition’s decision to pass the judicial reform laws, which are aimed at achieving a substantial change in the legal system. This ‘reform’ ignited heated debates, as a result of the sharp differences of opinion among the public in Israel. We assumed that people who voted for one of the parties that made up the coalition would express a positive attitude to the current changes and report good coping, compared to those who voted for one of the parties belonging to the opposition, who would express difficulties in coping with the changes, a higher level of distress, as well as a lower level of hope.

It is imperative to stress that though the background of this study is within the Israeli context, its importance lies beyond the specific social/political crisis in Israel. The study can be viewed as a glimpse into a dangerous process lurking at the doorstep of many democratic countries. One of the processes that characterize the current crisis may be termed “political radicalization”, which refers to the process according to which persons or groups adopt increasingly radical views in contradiction to a political, social, or religious status quo [10]. Among others, in this process, each political camp forms a conviction that they hold the moral high ground and typically dismisses the positions of the opposing camp [11]. Against this background, the current study is based on three repeated measurements: The same sample of participants completed a questionnaire three times. It examines the various effects of the political/societal crisis on the two main groups of the adult Jewish population: those who supported the coalition parties compared to those who supported the opposition parties in the November 2022 election. Furthermore, the present study focuses on the different effects of the social crisis on these two different groups, regarding three broad constructs as indicators of coping: (a) Societal (also terms `National`) resilience. (b) Supporting coping indicator: hope for a better future. (c) Suppressing coping indicators, including distress symptoms and a sense of danger.

The current study had three main goals: (a) To examine how the prolonged social/political crisis affects over time the two main groups of the Jewish population in Israel (coalition and opposition supporters) concerning distress symptoms, sense of danger, hope for a better future, and societal resilience. (b) to examine the differences in the aforementioned effects between coalition and opposition supporters. (c) to examine the differences over time (between the three measurements) for coalition and opposition supporters.

### Resilience

Resilience is a theoretical concept that has many definitions and has been extensively studied. Beyond the various definitions, it can be stated that resilience is the ability of humans to successfully cope with crises and disasters and to recover as much as possible afterward [12]. Many studies have examined resilience and various coping indicators, following human or natural-made disasters [e.g., 13]. However, the uniqueness of the current study is that it assesses the impact of elections, during an ongoing societal and political crisis on the resilience of the population, which was divided according to political beliefs.

#### Societal resilience (SR, also known as ‘national resilience’)

SR is vital for any society that must cope with threats to society as a whole or a large part of it. Some researchers explain the increase in research and debates concerning this concept by the multitude of crises that have threatened humans during the last decade, such as climate change, natural disasters, the Covid-19 pandemic, and disasters that result from human actions, such as war or terror attacks [14]. In a former study, we found that the concept of societal resilience is composed of four factors: trust in the government and its leaders, social integrity (also known as solidarity), a sense of attachment to the country (also known as patriotism), and trust in the state institutions [15]. Societal resilience is associated with coping indices during crises, such as armed conflict or the Covid-19 pandemic. For example, coping indices such as hope and morale were positively associated with societal resilience, while distress symptoms were negatively associated with societal resilience [e.g., 16–18]. The current study focuses on the SR of the State of Israel’s population in the context of the November 2022 elections and the subsequent change of government, that led to fundamental disagreements and an emerging social crisis.

### Coping indicators

Coping indicators are either positive predictors of the level of coping [19] or negative predictors of the level of coping [20]. Previous studies indicated that the higher the positive indicators are, the more they indicate successful coping, and vice versa [e.g., 21, 22]. We have examined in this study one positive coping indicator: hope for a better future [e.g., 23]. We have also examined two negative coping indicators: distress symptoms and a sense of danger.

#### Hope

The concept of hope has received many definitions in the professional literature [24]. Snyder, for example, defines hope as the expectation that something good will happen in the future [25]. Other researchers refer to hope as an emotional state [e.g., 26]. Beyond the various definitions, researchers assign great importance to hope as part of the successful management of varied stressful situations and effective coping with adversities [27]. Hope and resilience are related constructs as both involve a tendency towards an optimistic view in coping with adversities [28]. Previous studies have suggested that hope is the best predictor of SR [17].

#### Distress symptoms (also referred to as ‘psychological distress symptoms’)

Studies have found that different types of crises are associated with higher levels of distress symptoms. For example, a recent study reported significant distress symptoms that were manifested in wide-world large samples in response to the Super Typhoon in the Philippines, COVID-19 in China and the UK, and terror attacks in UK, USA, and France [29]. Distress symptoms examined in the current study included anxiety and depressive symptoms, which are among the most prominent responses of people, resulting from stressful situations as well as various threats [30].

#### Sense of danger

Sense of danger [based on 31] presents the extent to which the individual perceives the current situation as dangerous for oneself or family members. The level of sense of danger is used as a negative indicator of the individual’s coping capacity [32].

### Research hypotheses

Based on the background described above, and assuming that voters for the opposition perceived the changes that the new government initiated as a crisis that may affect them personally, we formulated the following hypotheses:

1. The level of societal resilience will significantly increase among the coalition voters and significantly decrease among the opposition voters, along the two post-election measurements compared with the measurement that was taken before the election.
2. The level of hope will significantly increase among the coalition voters and significantly decrease among the opposition voters along the three measurements.
3. The level of the coping suppressing indicators (distress symptoms and sense of danger) will significantly decrease among the coalition voters and significantly increase among the opposition voters along the three measurements.

## Method

### Participants

This study is based on three repeated measurements in which the same respondents participated three times. The first measurement was conducted on October 11–18, 2022, a short time before the November 1^st^ election day. The second measurement was conducted two and a half months after the elections, on February 18–23, 2023, after the establishment of the right-wing government. This period was in the midst of the "judicial reform" crisis, during the week in which some of the new laws were debated in the parliament’s Constitution Committee and were brought to a vote. A third measurement was conducted on August 7–10, 2023, after the first law was enacted by the Israeli parliament (Knesset). Eligible to participate in the study were adults over 18 years old. The study is based only on the answers of the respondents who completed the questionnaire in all three measurements (N = 895). The respondents were classified into two groups, based on their report (in T2) regarding the party they voted for in the November 1^st^ elections (see Table 1). Thus, the findings presented in this paper examine the differences between respondents who voted for one of the parties that eventually composed the coalition (n = 431) and respondents who voted for one of the parties that belonged to the (new) opposition (n = 354). Respondents who voted for a party that did not pass the electoral threshold, or voted for an Arab party, were not included in this analysis (n = 110), as it is not clear whether they consider themselves as belonging to one of the above political factions. Therefore, the final entire sample is composed of 785 respondents. The questionnaire was distributed through an Internet panel (Sekernet company; https://sekernet.co.il/) that includes over 65,000 people, representing the various groups and sectors of the population. A stratified sampling method was employed, aligned with the data of the Israeli Central Bureau of Statistics. The questionnaire was approved by the Ethics Committee of Tel Aviv University and all the participants expressed their written informed consent.

**Table 1:** Demographic characteristics of the whole sample (N = 785) and the two political blocs ‐ coalition voters (n = 431) and opposition voters (n = 354).

Variable	Group	Whole sample (N = 785) Number (%)	Coalition Voters (n = 431) Number (%)	Opposition Voters (n = 354) Number (%)	T-test or Chi-square comparisons
Age	18–30	147 (18.7)	112 (26.0)	35 (9.9)	
	31–40	153 (19.5)	89 (20.6)	64 (18.1)	
	41–50	164 (20.9)	86 (20.0)	78 (22.0)	
	51–60	148 (18.9)	78 (18.3)	69 (19.5)	
	61–82	173 (22.0)	66 (15.1)	108 (30.5)	
		**Average (S.D)** 46.68 (15.40)	**Average (S.D)** 43.27 (15.13)	**Average (S.D)** 50.84 (14.71)	t = 7.058*; Cohen’s d = 0.51
Gender	1. Men	423 (53.1)	229 (53.1)	195 (55.1)	***χ***^2^ = 1.48 (NS)
	2. Women	362 (46.9)	202 (46.9)	159 (44.9)	
Degree of religiosity	1. Secular	360 (45.9)	97 (22.5)	263 (74.3)	
	2. Traditional	256 (32.6)	177 (41.1)	79 (22.3)	
	3. Religious	102 (13.0)	94 (21.8)	8 (2.3)	
	4. Very religious	67 (8.5)	63 (14.6)	4 (1.1)	
		**Mode:** 1	**Mode:** 2	**Mode:** 1	***χ***^2^ = 233.216*
Average family income relative to the Israeli average	1. Much lower	247 (31.5)	166 (38.5)	81 (22.9)	
	2. Lower than average	171 (21.8)	97 (22.5)	74 (20.9)	
	3. About the same as average	211 (26.8)	99 (23.0)	112 (31.6)	
	4. Higher than average	117 (14.9)	55 (12.8)	62 (17.5)	
	5. Much higher	39 (5.0)	14 (3.2)	25 (7.1)	
		**Mode:** 1	**Mode:** 1	**Mode:** 3	***χ***^2^ = 29.397*
Political stances	1. Very left	3 (0.4)	0 (0.0)	3 (0.8)	
	2. Left	59 (7.5)	2 (0.5)	57 (16.1)	
	3. Center	267 (34.0)	59 (13.7)	208 (58.8)	
	4. Right	363 (46.2)	281 (65.2)	82 (23.2)	
	5. Very right	93 (11.8)	89 (20.6)	4 (1.1)	
		**Mode:** 4	**Mode:** 4	**Mode:** 3	***χ***^2^ = 319.726*
Education level	1. Elementary school	15 (1.9)	14 (3.2)	1 (0.3)	
	2. High school	171 (21.8)	123 (28.5)	48 (13.6)	
	3. Partial academic	217 (27.6)	119 (27.6)	98 (27.7)	
	4. Bachelor’s degree	234 (29.8)	115 (26.8)	119 (33.6)	
	5. Master’s degree +	148 (18.9)	60 (13.9)	88 (24.8)	
		**Mode:** 4	**Mode:** 2	**Mode:** 4	***χ***^2^ = 44.434*
Party voted for in the November 2022 election.	1. Likud		231 (29.4)		
	3. Religious Zionism		115 (14.6)		
	5. Shas		39 (5.0)		
	6. Torah Judaism		46 (5.9)		
	2. There is a future			188 (23.9)	
	4. The State Camp			87 (11.1)	
	7. Yisrael Beiteinu			35 (4.5)	
	10. Labor Party			44 (5.6)	

p<0.0001

### Study tools

All the scales included in the study were used in previous studies and showed good validity and reliability. The measurement scales included:

**Societal resilience** [33]. This scale includes 15 items, e.g., “I love my country and am proud of it”. The scale for each item ranges from 1 = `strongly disagree`to 6 = `strongly agree`, thus a higher mean score indicates higher societal resilience. The current study’s Cronbach’s alpha reliability of this measure was high in all measurements (α = .90 for T1 and α = .87 for both T2 and T3).

#### Hope

This scale is based on previous scales [34, 35]. The scale includes five items, e.g., “I will emerge strengthened from the current crisis”. The scale ranged from 1 = `very little hope`to 5 = `very much hope`, thus a higher mean score indicates a higher level of hope. The current study’s Cronbach’s alpha reliability of this measure was high in all measurements (α = .91, α = .93, and α = .95, for T1, T2, and T3, respectively).

**Distress** symptoms (This scale includes items concerning anxiety and depression symptoms; BSI, [36]). The tool presents eight items describing different symptoms (four for anxiety symptoms, for example, “in the past days, how much were you distressed by nervousness; and four for depressive symptoms, for example, “in the past days, how much were you distressed by feeling lonely”) ranging from 1 ’not at all`to 5 = `to a very large extent, thus a higher mean score indicates higher distress levels. The Cronbach’s alpha reliability of this measure in the current study was high in all measurements (α = .92, for both T1 and T2, and α = .93, for T3).

**Sense of Danger** [31] includes seven items, e.g., “To what extent do you feel that your life is in danger?”. The scale ranges from 1 = `not at all’ to 5 = `to a very large extent`, thus a higher mean score indicates higher levels of sense of danger. The Cronbach’s alpha reliability of this measure in the current study was good in all measurements (α = .84, α = .86, and α = .88, for T1, T2, and T3, respectively).

**Demographic details**: Age, gender, religiosity (which refers to the degree of religious life style, average family income, political attitudes, and political orientation (see a distribution of demographic characteristics for the whole sample and categorized according to the two political blocs, coalition and opposition voters, in Table 1).

### Statistical analyses

The data were analyzed as two-factor repeated-measures ANOVA, with a mixed-design General Linear Model (GLM) procedure using SPSS software (version 28) on all of the relevant measurements: SR, hope, distress symptoms, and sense of danger. The between-subjects variable was the political affiliation of the voter’s group (to be termed “bloc”), which reflects whether the participant voted for a party belonging to the (new) coalition or the (new) opposition, based on his/her answer at T2 (post-election). The repeated variable was the measurement time (T1, T2, and T3).

## Results

### Demographic differences

Comparing participants’ demographic characteristics indicated five significant differences between the coalition and opposition voters: The coalition voters are significantly younger, less educated, more religious, reported a significantly lower level of income, and possess more right-wing political attitudes, compared with the opposition voters (Table 1).

### GLM mixed design analyses–repeated measure ANCOVAs

Four different GLM mixed design analyses (repeated measure ANCOVAs) with the variable political bloc as the between-subjects variable (coalition voters vs. opposition voters), the variable measurement time (T1, T2, and T3) as the repeated variable, with age, family income, education level, and level of religiosity as covariates, were conducted, one analysis for each measurement scale (i.e., mean scores of SR, hope, distress symptoms, and sense of danger). As can be seen in Table 2, all the interactions between the two variables were significant with medium to large effect sizes.

**Table 2:** Repeated measures ANCOVAs (measurement time × political bloc) of all measurements with covariates (age, family income, education level, and level of religiosity).

Variable	Effect	df	F	p	ηp^2^
SR	Bloc	1, 778	42.027	<0.0001	0.051
	Measurement time	2, 778	0.112	0.894	0.000
	Bloc × Measurement time	2, 778	67.545	<0.0001	0.080
Hope	Bloc	1, 778	49.389	<0.0001	0.060
	Measurement time	2, 778	2.558	0.078	0.003
	Bloc × Measurement time	2, 778	22.631	<0.0001	0.028
Distress symptoms	Bloc	1, 778	10.250	<0.01	0.013
	Measurement time	2, 778	1.298	0.237	0.002
	Bloc × Measurement time	2, 778	21.080	<0.0001	0.026
Sense of danger	Bloc	1, 778	9.138	<0.003	0.012
	Measurement time	2, 778	2.976	0.051	0.004
	Bloc × Measurement time	2, 778	65.198	<0.0001	0.077

**Fig 1:**
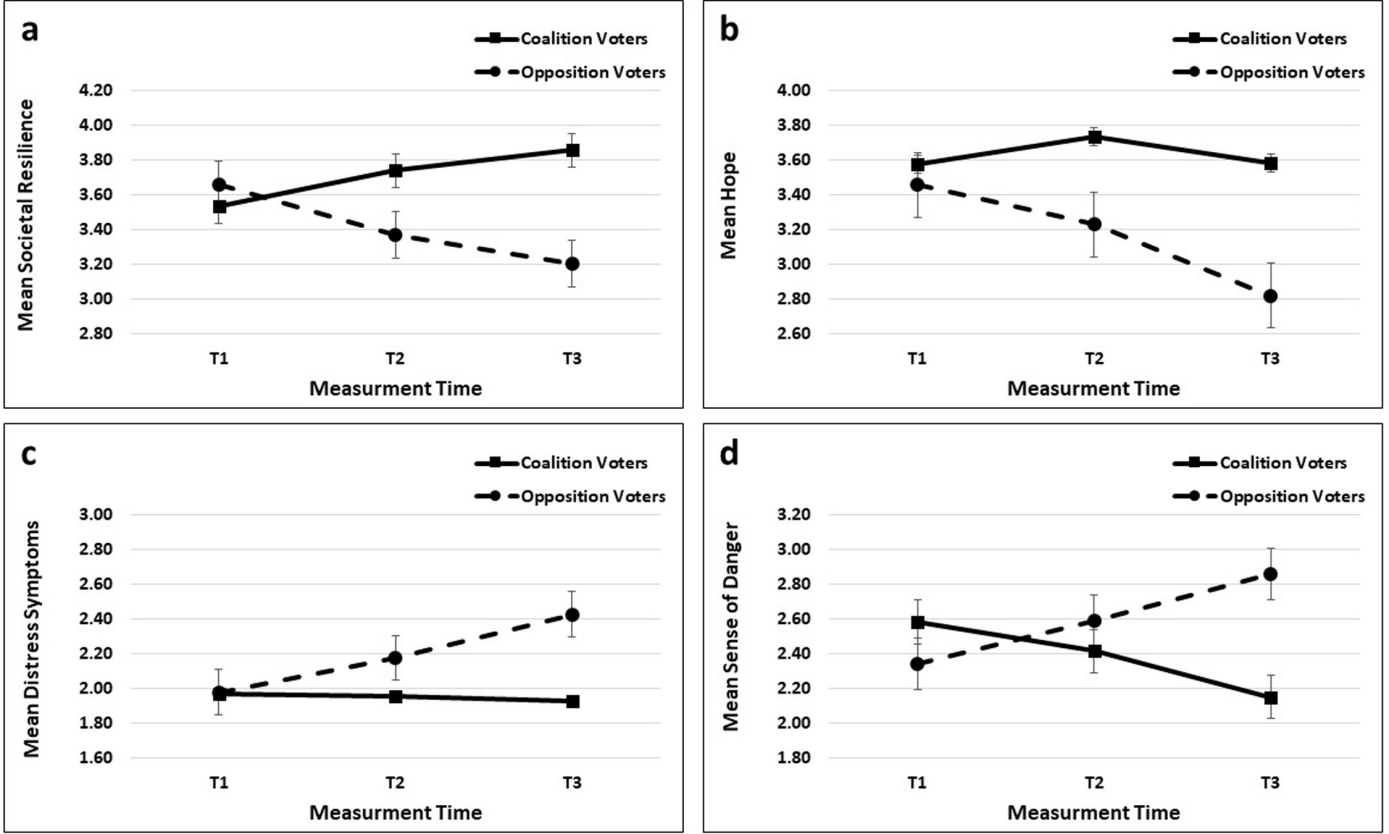
Two-way interaction between political bloc (coalition vs. opposition voters) and measurement time (T1 –before the November 2022 election; T2 –two and a half months after the election, T3 –nine months after the election). a) Societal resilience (SR); b) Hope; c) Distress symptoms; d) Sense of danger.

Fig 1 presents the four interactions and indicates the time measurement differences, as well as the differences between the two blocs in each of the measurements. Table 3 presents the size of the extending gap between the two groups. It can be seen that: (a) SR: Fig 1A shows that the SR of the two different political bloc groups was significantly different in the first measurement with a slightly higher mean SR among the opposition voters compared with that of the coalition voters (p<0.05). However, in the two measurements that were conducted following the election, the pattern was inverted, presenting significantly higher SR among the coalition voters and a widening gap from T2 and T3 between the groups (p<0.05). (b) Hope: Looking at Fig 1B, it can be concluded that the level of hope of the coalition voters is significantly higher compared with that of the opposition voters during all measurement times (p<0.05). In addition, while the coalition voters’ level of hope significantly increased from T1 to T2, it decreased back to its former level in T3. In contrast, the level of hope of the opposition voters significantly and quite sharply decreased from one measurement to the other, yielding, again, a widening gap between the two groups (all differences were significant at p<0.05). (c) Distress symptoms: Fig 1C shows different patterns between the two groups concerning the distress symptoms scale. At T1 there was no difference between the two groups, presenting quite low levels of distress symptoms among all the participants. However, while the symptoms of the coalition voters did not change over the three measurements, those of the opposition voters increased significantly from one measurement to the other (p<0.05), resulting, again, in a widening gap between the two groups from T2 to T3. (d) Sense of danger: A similar yet opposite direction pattern can be seen in Fig 1D, regarding the sense of danger scale. In T1 the mean sense of danger was slightly but significantly higher among the coalition voters compared with that of the opposition voters (p<0.05). Yet, the pattern was reversed in T2 and T3, with a significantly much higher sense of danger for the opposition voters and a significantly increased gap from T2 to T3 (p<0.05).

**Table 3:** The size of the gap between the two political blocs at the three measurements (coalition minus opposition).

Variable	T1	T2	T3
SR	-0.13	0.37	0.65
Hope	0.12	0.51	0.77
Distress symptoms	-0.01	-0.22	-0.50
Sense of danger	0.24	-0.17	-0.71

## Discussion

The current study examined the impact of the November 2022 election on a longitudinal sample of the Jewish adult population in Israel, which was divided into two groups: coalition and opposition voters. The first measurement was conducted a few weeks before the election. The second measurement was conducted two and a half months after the election, during an acute social/political crisis that emerged resulting from the new government’s attempt to enact laws constituting a "judicial reform" which is viewed by some citizens as a threat to the democracy of the state of Israel. The third measurement was conducted on August 7–10, 2023, after more than 35 weeks of mass demonstrations nationwide. In this study, we examined the impact of the social/political crisis on two groups of Israeli Jewish citizens regarding societal resilience and indicators of coping ‐ level of hope, distress symptoms, and sense of danger.

The salient findings of the current study point to a substantial variability between the two groups of voters regarding the four research variables. In addition, the results show that the psychological endurance of those who voted for the opposition was negatively impacted, when comparing their pre-election levels (T1) to their post-election levels T2 and T3, which is apparent for all scales that were measured in the current study. In contrast, those who voted for the coalition parties showed improvement in several variables (SR, hope, and sense of danger) but did not improve concerning their distress symptoms.

Overall, these findings support our three hypotheses, suggesting that for voters of the opposition, the political conflict and its consequences brought about a deterioration of their psychological state. In contrast, for the voters of the coalition, the elections led to an improvement in their psychological state concerning most of the variables assessed in the current study, apart from distress symptoms. These results may superficially resemble the winner-loser gap found in studies on the variable of ’satisfaction with democracy’. This effect typically shows an increase in satisfaction with democracy among the winners, while the satisfaction of the losers tends to decrease [see review in 37]. However, two important issues differentiate the current findings from being interpreted as a winner-loser gap. First, the winner-loser gap typically addresses attitudes toward democracy, whereas this study explored societal resilience, hope, feelings of distress, and sense of danger, which are all psychological constructs rather than attitudinal measures. Second, it is important to emphasize that the current measurements were taken during a prolonged social and political crisis characterized by large-scale, weekly protests against the government, involving a significant portion of the population. Consequently, the observed widening gap between the two groups of voters from one measurement to the next suggests that the current data cannot be interpreted as a reflection of the winner-loser gap, which tends to remain stable or decrease [37]. Instead, we claim that the current results indicate a genuine psychological deterioration among opposition voters, while coalition voters experienced an improvement in their psychological state.

Potential explanations of these results are as follows: First, previous studies that have focused on “political radicalization”[e.g., 10, 38] have shown that increasing gaps between political groups and rejecting the other(s) is an effective way to lead a political struggle [39]. The definition of this concept implies the deviation of political attitudes away from the center towards ideological extremes [40]. The differences between the two voter groups in the current study may be primarily the result of the political radicalization and polarization process based on widening the gaps between groups as part of the active building of political power. The importance of the present study stems from the fact that it reveals the results of political radicalization and its effect on the population’s psychological well-being within a relatively short period, before and after an election round. Though the current study took place within the framework of a specific country (Israel) we suggest that this is of global importance, because the underlying process is not unique only to one society. Many countries are currently facing acute political crises in which the country is undergoing political radicalization, and the different camps, or groups, are unwilling to accept the possibility of a compromise that requires a partial agreement with the attitudes of the other faction [11, 41].

Second, the demographic differences between the two voters’ groups (such as age, level of religiosity, education level, and level of income) may contribute to the differences in their perceptions regarding the potential consequences of the present societal crisis. It should be noted that the ANCOVA analyses revealed differences between the groups even when demographic variables were included as covariates. Nevertheless, the differences between the two groups may represent socio-cultural differences that have always existed in Israeli society, and the results of the November 2022 election may only highlight them. Indeed, previous studies have found clear associations between political positions and attitudes concerning crisis management, for example during the COVID-19 pandemic [17, 42]. However, these explanations necessitate further research support, including from other cultures and diverse cultural contexts.

Third (but related to the second explanation), it is possible that at least some of the coalition voters, who reported that they are religious or very religious, perceive the political change following the election as an intervention that expresses God’s will, and therefore see the results as meaningful beyond rational thinking [43]. They thus continue to support the “judicial reform” that the government promotes and view the government’s actions with a positive attitude, even when it is in contrast to the economic analyses of experts, as published in many economic journals [44].

Another important issue that emerges from the current study is of more methodological concern. Instead of relying on the overall sample mean, representing the central tendency of the entire sample as is customary in many studies, the current study examined the distributions and differences between the variables according to the classification of the target population into two groups, coalition and opposition voters. It should be noted that if the data were analyzed as a whole, based on the general average, important insights concerning the impact of the election on the population’s psychological state may have been overlooked. In that case, it might have been concluded that the election and the protests that followed after the government actions concerning the "judicial reform" did not affect the population’s psychosocial responses at all, because when averaging the entire sample most of the differences, which showed contrasting directions among the two voters’ groups, would be eliminated. Yet, as can be seen in the current study, the election had a great effect on both groups, but in different directions. Thus, this finding demonstrates that the mean of the overall sample may not be an appropriate measurement in studies dealing with a society that struggles with political/social radicalization.

### Limitations

Several limitations of the present study should be noted: (a) Though relying on an Internet panel survey that represents the diverse sections of Israeli society, it cannot be stated unequivocally that the sample represents the adult population in Israel. (b) No similar study was found, and therefore it is not possible to compare the current study results with other studies, and they should be treated with caution. (c) The results of the present study do not allow us to determine whether and to what extent the differences between the two voter groups represent differences between the group’s characteristics or are the result of differences in their opinions.

## Conclusions

Based on the differences between the coalition and opposition voters regarding the examined variables, it can be concluded that the continued social/political conflict in Israel is multidimensional and includes diverse areas such as values, perspectives, and supporting as well as suppressing coping indicators. There are notable differences related to societal resilience, hope, distress symptoms, and feelings of danger between the coalition and opposition voters. Despite the advantages of a longitudinal study, the current research cannot reveal what are the causes of the variability between the voters’ groups, nor how and when they have been developed. It is therefore highly recommended to conduct similar studies before and after crucial elections in other countries, to examine whether similar results will be found and whether the current findings can be generalized to other societies as well.
