# Correction: Impact of prolonged social crisis on resilience and coping indicators

**DOI:** 10.1371/journal.pone.0329662

**Published:** 2025-08-04

**Authors:** Hadas Marciano, Shaul Kimhi, Yohanan Eshel, Bruria Adini

There are errors in the author affiliations. The correct affiliations are as follows:

Hadas Marciano^1,2^, Shaul Kimhi^3^, Yohanan Eshel^1,4^, Bruria Adini^3,5^

1 Stress and Resilience Research Center, Tel-Hai College, Tel Hai, Israel, 2 Institute of Information Processing and Decision Making (IIPDM), University of Haifa, Haifa, Israel, 3 ResWell Research Collaboration, Tel Aviv University, Tel Aviv, Israel, 4 Psychology Department, University of Haifa, Haifa, Israel, 5 Head of the Department of Emergency Management and Disaster Management, School of Public Health, Gray Faculty of Medical and Health Sciences, Tel Aviv University, Tel Aviv, Israel.
